# Proton-Coupled Electron Transfer from the Hydride Perspective: Resolving Metal Oxidation and Protonation in a Hydridocarbonyl Complex by 2D-IR Spectroelectrochemistry

**DOI:** 10.1021/acs.jpclett.5c03977

**Published:** 2026-02-25

**Authors:** Ricardo J. Fernández-Terán, Iona I. Ivalo, Dimitri Chekulaev, Julia A. Weinstein

**Affiliations:** † Department of Physical Chemistry, 27212 University of Geneva, CH-1205, Geneva, Switzerland; ‡ Department of Chemistry, 7315 University of Sheffield, Sheffield S3 7HF, United Kingdom

## Abstract

Proton-coupled electron transfer (PCET) is a fundamental process in energy conversion and catalysis, yet direct spectroscopic characterization of metal hydride intermediates remains challenging. Here, we employ ultrafast two-dimensional infrared spectroelectrochemistry (2D-IR-SEC) to investigate the structural and vibrational dynamics of a model hydridocarbonyl complex, [HIr^I^(CO)­(PPh_3_)_3_] (**H1**), during sequential oxidation and PCET reactions. The strong anharmonic coupling between the Ir–H and Ir­(C≡O) stretching modes enables simultaneous monitoring of the metal oxidation state and hydride protonation state in real time. Spectroelectrochemical studies reveal that the first oxidation reversibly generates a stable 17-electron species. The second oxidation, in contrast, triggers irreversible PCET-driven deprotonation. 2D-IR-SEC spectra of the singly oxidized species show motional narrowing of the band with a stronger Ir–H character, reflecting altered solvation dynamics upon oxidation. This work thus establishes 2D-IR-SEC as a powerful tool for resolving coupled electron and proton transfer events in metal hydrides, with implications for the design of PCET-mediated catalysts.

## Graphical abstract

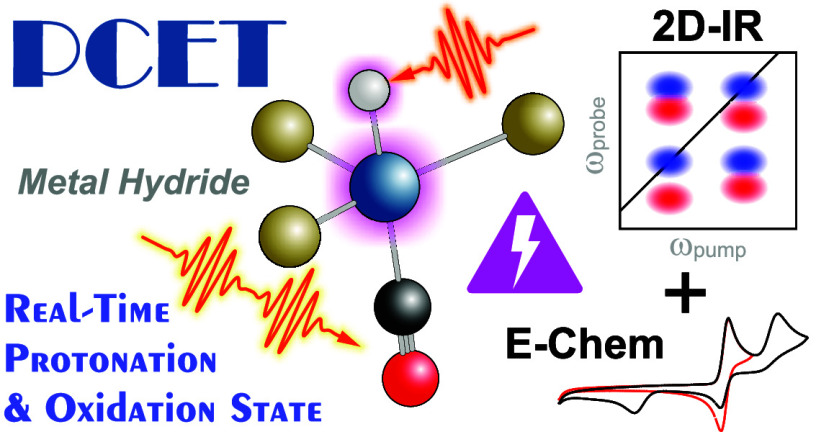

Proton-coupled electron transfer (PCET) is a fundamental process in nature. In photosynthesis, it plays a central role in maintaining charge neutrality in the reaction center and across cellular membranes.^1^ PCET is also involved in CO_2_ reduction and the production of hydrogen and other solar fuels. These catalytic processes involve coupled proton and electron transfer steps that influence each other.^2,3^

The M–H bond in metal hydride complexes is of particular interest because metal hydrides can participate directly in PCET reactions, acting as either hydride, proton or hydrogen atom donors.^4^ Transition metal hydride complexes have been widely used as models to study PCET reactions with external oxidants and internal or external bases, further highlighting their relevance in this context.^5–8^ To improve the efficiency of energy conversion and solar fuel production, a deeper understanding of the PCET reactivity of metal hydrides is essential. However, most studies on transition metal hydride complexes evaluate the PCET dynamics indirectly—from the perspective of either the oxidant or base—and spectroscopic methods directly addressing the metal hydride complexes are desired.

Weak M–H stretching vibrations can gain intensity through electronic communication with a *trans*-carbonyl ligand (mediated by metal and ligand orbitals) and anharmonic coupling with the M­(C≡O) vibrations, as demonstrated in our previous work using ultrafast two-dimensional infrared spectroscopy (2D-IR).^9^ Dihydrides with a *trans* H–M–H arrangement of the hydride ligands have also shown strong mutual enhancement of the M–H vibrations.^10–12^ 2D-IR provides insights beyond linear absorption spectroscopy, revealing vibrational couplings, anharmonicities, population dynamics (vibrational lifetimes), and spectral diffusion (related to solvent fluctuations).^13^ Recently, we used 2D-IR spectroscopy to study a series of Pt­(II) bis-acetylide donor–bridge–acceptor complexes, showing that the *trans* acetylides decouple upon isotopic labeling on one side.^14^

Herein, we study the hydridocarbonyl complex [HIr^I^(CO)­(PPh_3_)_3_] (**H1**), as a model system to investigate changes in the structural and vibrational dynamics following electron transfer and subsequent proton-coupled electron transfer reactions, as depicted in Scheme 1. Using ultrafast 2D-IR spectroelectrochemistry (2D-IR-SEC) and density functional theory (DFT) calculations, we elucidate the nature, properties and identities of the observed species.

**Scheme 1:**
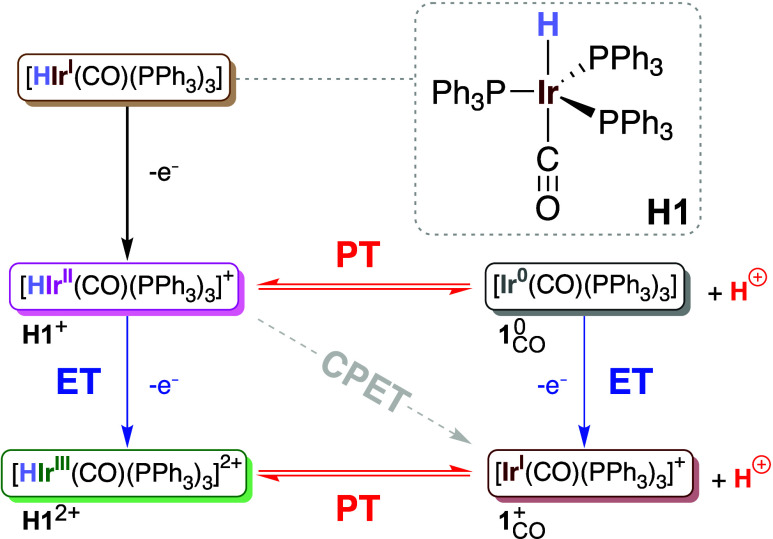
Structure of the Studied Complex (**H1**) and Possible PCET Pathways after the First Oxidation^a^

a ET/PT = electron/proton transfer; CPET = Concerted proton–electron tunnelling.

In complexes with both hydride and carbonyl ligands, the M­(C≡O) stretching bands report on the oxidation state of the metal center, while the M–H stretching bands provide information about the hydride ligand. Anharmonic coupling between these vibrational modes delocalizes the M–H and M­(C≡O) stretching vibrations, enabling the determination of both the oxidation state [via M­(C≡O) frequencies] and protonation state (via 2D-IR cross peaks) during PCET reactions. Thus, 2D-IR can unambiguously reveal the oxidation state of the metal, and the status of the hydride ligand in real time.

Cyclic voltammograms of **H1** (with 5 eq. PPh_3_)^15^ in DCM (Figure 1) show a first, reversible oxidation event with *E*_1/2_^ox1^ = – 440 mV vs Fc^+/0^. Further scanning the potential in the oxidative direction reveals a second, irreversible oxidation process at *E*_pa_^ox2^ = +130 mV vs Fc^+/0^. At the same time, a new reduction event is observed at *E*_pc_^red^ = – 1.42 V vs Fc^+/0^ on the reverse scan when the potential is scanned past the first oxidation event. The irreversible oxidation of PPh_3_ occurs at much higher potentials, with *E*_pa_^PPh_3_^ = +810 mV vs Fc^+/0^.

**Figure 1:**
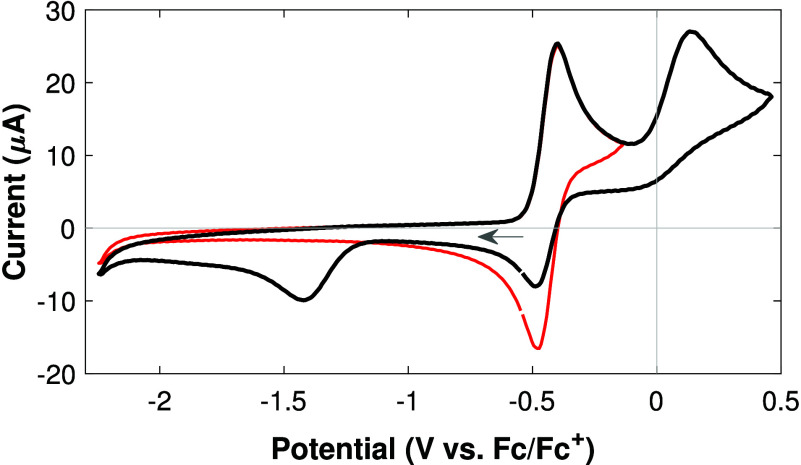
Cyclic voltammograms of **H1** (2 mM, with 5 eq. PPh_3_) in 0.4 M [Bu_4_N]­[PF_6_] in DCM, scanned up to the 1st (red trace) and 2nd oxidation (black trace), at 100 mV s^–1^. Both plots show the 2nd CV cycle. The feature at ca. – 1.5 V is absent on the first cycle or when scanning below the 2nd oxidation. See text for discussion.

The first oxidation of **H1** leads to the stable [HIr^II^(CO)­(PPh_3_)_3_]^+^ 17-electron cationic species (**H1** ^+^). This process is fully electrochemically reversible and diffusion-controlled, as demonstrated by cyclic voltammograms obtained at different scan rates (see ), in agreement with previous reports.^16,17^

The second oxidation leads to deprotonation of the complex via a proton-coupled electron transfer mechanism, as shown in Scheme 1, and in line with previous reports.^16,17^ The electrochemically generated **H1** ^+^ species may also disproportionate—on a longer time scale—and evolve hydrogen irreversibly, according to eq 1 (as reported in refs 17 and 18):

1 $$2{[{\mathrm{HIr}}^{\mathrm{II}}\left(\mathrm{CO}\right)\left({\mathrm{PPh}}_{3}{)}_{3}\right]}^{+}\to 2{[{\mathrm{Ir}}^{I}\left(\mathrm{CO}\right)\left({\mathrm{PPh}}_{3}{)}_{3}\right]}^{+}+H_{2}↑$$

While eq 1 represents the net stoichiometric change yielding [Ir^I^(CO)­(PPh_3_)_3_]^+^ (**1**_CO_^+^), the exact mechanism of this reaction may involve a mixture of deprotonation and disproportionation of **H1** ^+^, further complicated by potential side reactions involving hydrogen/protons at the Pt electrode.^16–18^

Steady-state SEC experiments in both the UV/vis and mid-IR spectral domains (Scheme 2) reveal distinct changes as a function of the applied redox potential. Table 1 summarizes the experimental and calculated absorption bands for all species discussed in this work.

**Scheme 2:**
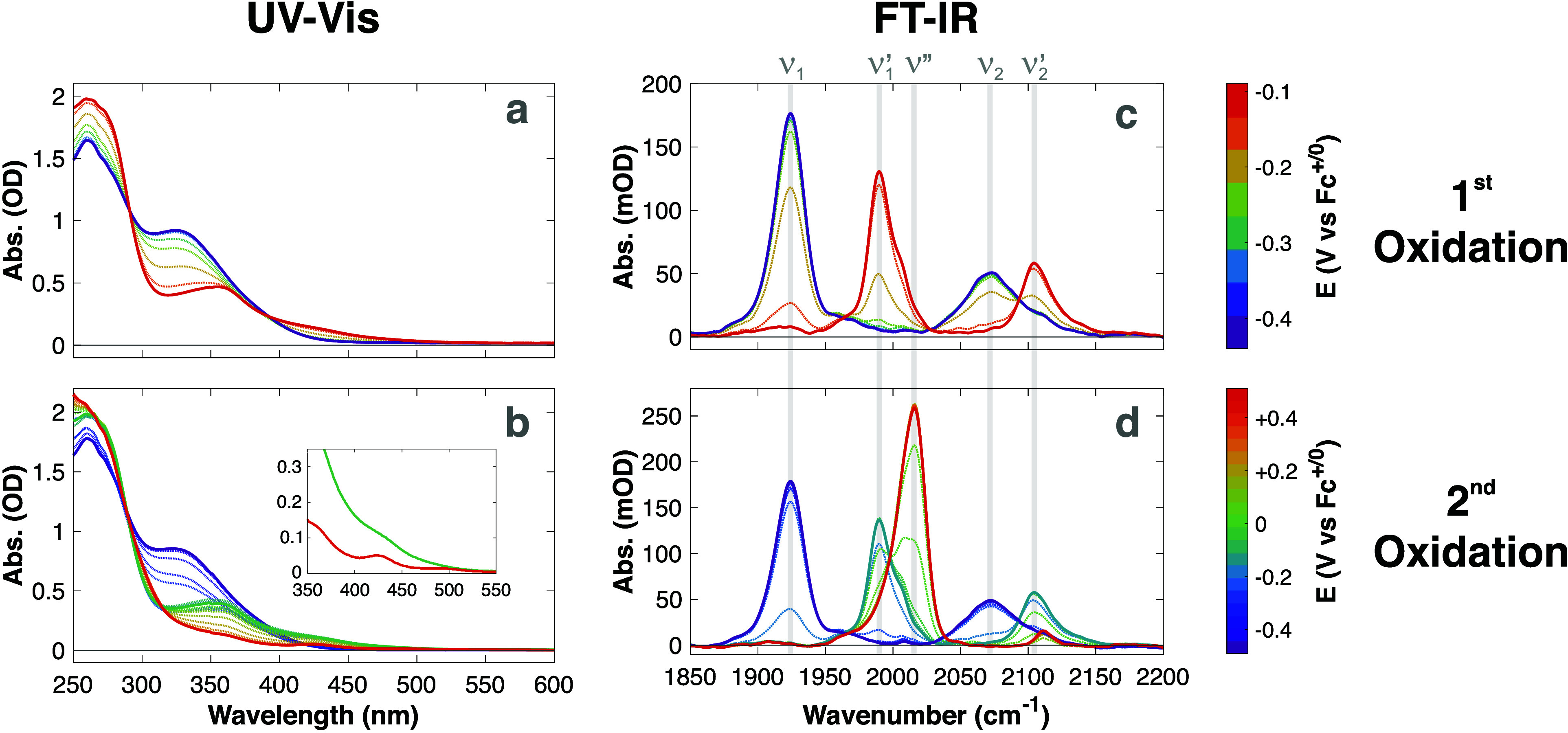
Steady-State Spectroelectrochemical Studies of **H1** in 0.4 M [Bu_4_N]­[PF_6_] in DCM^a^

a Potential-dependent UV/Vis (*A–B*) and FT-IR spectra (*C–D*) are shown, corresponding to the first (*top row*) and second (*bottom row*) oxidation processes. The labelled bands in the IR-SEC spectra are discussed in the main text and in Table 1. The inset in *B* emphasises the differences in the UV/Vis absorption spectra after the first (green) and second (red) oxidations, respectively.

**Table 1:** Summary of the Experimental and DFT-Calculated Spectroscopic Properties of the Complexes Studied in This Work

		FT-IR SEC (cm^–1^)^a^	UV/vis SEC (nm)^a^
**H1**	*Exp.*:	1924 (ν_1_), 2072 (ν_2_)	260, 322
	*Calc.*:^c^	1928 (ν_1_), 2069 (ν_2_)	
**H1** ^+^	*Exp.*:	1990 (ν_1_^′^), 2105 (ν_2_^′^), 2006^e^	260, 356, ca. 430^e^
	*Calc.*:^c^	1993 (ν_1_^′^), 2113 (ν_2_^′^)	
**H1** ^2+^	*Exp.*:	n/d^d^ [lit.: 2068 (ν_1_^″^), 2176 (ν_2_^″^)]^b^	n/d^d^
	*Calc.*:^c^	2074 (ν_1_^″^), 2219 (ν_2_^″^)	
**1**_CO_^+^	*Exp.*:	2016 (ν″)	250, 360^e^, 425, 495
	*Calc.*:^c^	2025 (ν″)	
**1**_CO_^0^	*Exp.*:	n/d^d^	n/d^d^
	*Calc.*:^c^	1902	

a From this work, unless otherwise stated.

b From ref 17. (conditions: DCM, 0.2 M [Bu_4_N]­[PF_6_], – 45 °C).

c Harmonic frequencies, scaled by 0.971; see for further details.

d Not detected under our experimental conditions (DCM, 0.4 M [Bu_4_N]­[PF_6_], OTTLE cell, 25 °C).

e Shoulder or unresolved absorption band.

The interpretation of these spectral changes is not generally unambiguous. As is often the case, multiple species could be present, and it is only with the help of multidimensional spectroscopic techniques such as 2D-IR that this ambiguity can be lifted. 2D-IR spectra can reveal information which is not accessible to linear IR spectroscopy, such as vibrational couplings, anharmonicities and band inhomogeneities.^13^

Pioneering work by Bredenbeck and co-workers,^19^ Kubarych and co-workers,^20,21^ and Oppelt, Hamm and co-workers^22,23^ has illustrated the use of 2D-IR-SEC to study oxidation-state-dependent vibrational dynamics. In this work, we employ pulse-shaping-based^24–26^ 2D-IR-SEC in transmission geometry, using an optically transparent thin layer electrochemical cell (OTTLE cell).^27^

We have previously examined the vibrational spectrum of **H1** (Figure 2),^9^ which shows two distinct IR absorption bands at 1924 and 2072 cm^–1^. The lower frequency mode (ν_1_) has a predominant Ir­(C≡O) character (ca. 80%), while the ν_2_ mode contains a larger participation factor of the Ir–H stretching mode (∼70%). These modes are strongly coupled and delocalized, as the Ir­(C≡O) and Ir–H vibrations mix into ν_1_ and ν_2_.

**Figure 2:**
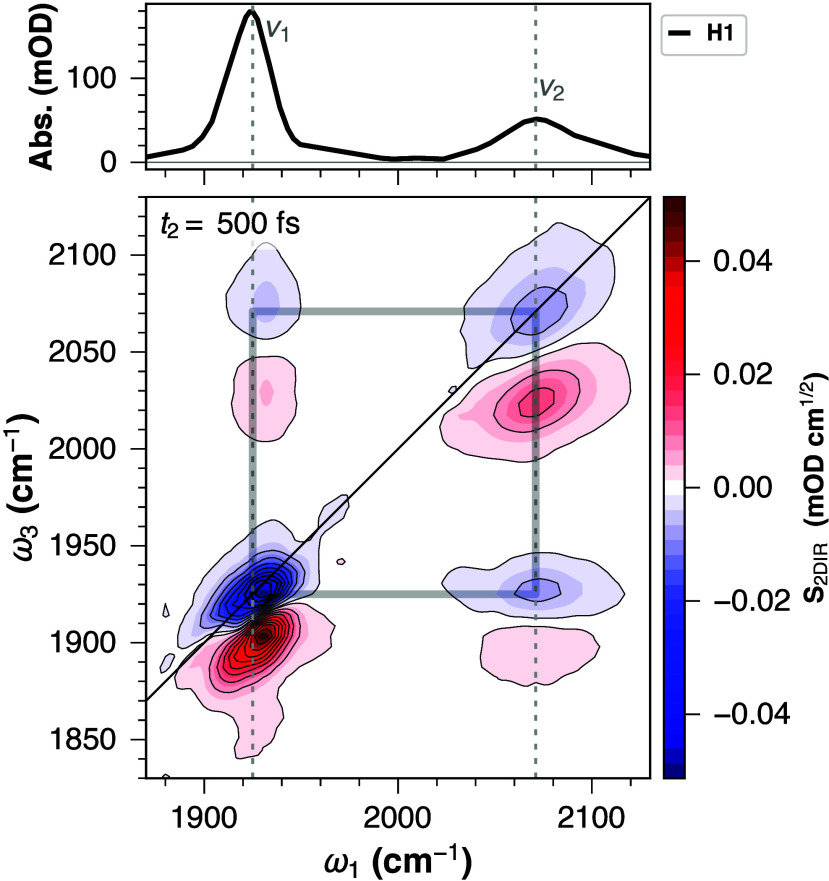
FT-IR (*top*) and 2D-IR (*bottom*) spectra of **H1** in DCM (with excess PPh_3_). The vertical dashed lines connect the absorption and 2D-IR spectral features, discussed in the text. A square is used to guide the eye to the coupled ν_1_ and ν_2_ vibrations.

Upon the first oxidation, we observe a systematic blue shift of these IR absorption bands by ca. 66 and 33 cm^–1^ (for ν_1_ and ν_2_, respectively; which we now denominate as ν_*x*_^′^ for the **H1** ^+^ complex). The relative intensities of ν_1_^′^ and ν_2_^′^ remain largely unchanged, however the line shapes appear different. An additional shoulder at 2006 cm^–1^ is observed, whose origin and nature are not fully understood.

The 2D-IR-SEC spectrum of **H1** ^+^ (Figure 3) reveals a similar structure as was observed for the parent complex (**H1**), with an additional band (ν″) whose origin we tentatively attribute to formation of the **1**_CO_^+^ species (via disproportionation, eq 1). We base this later assignment on the diagonal anharmonicity, and longer vibrational lifetime compared to ν_1_^′^ and ν_2_^′^, as discussed below. The two-dimensional line shape of the ν_2_^′^ band appears motionally narrowed, while the inhomogeneous contribution clearly persists beyond the vibrational lifetime (*vide infra*).

**Figure 3:**
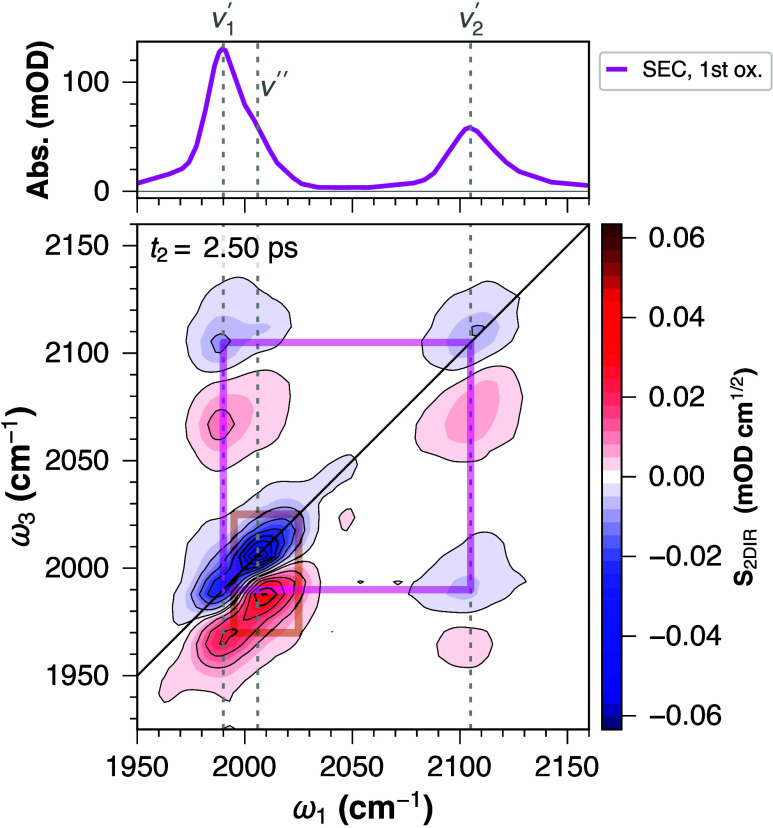
FT-IR SEC (*top*) and 2D-IR-SEC (*bottom*) spectra of **H1** ^+^ (at – 380 mV vs Fc^+/0^ in DCM). The vertical dashed lines connect the absorption and 2D-IR-SEC spectral features, discussed in the text. Squares are used to guide the eye to the coupled ν_1_^′^ and ν_2_^′^ vibrations (magenta), and to the additional band ν″ (brown).

The apparent diagonal anharmonicities (Δ_*jj*_, extracted by 2D Gaussian fitting as described in ref 28) of the ν_1_^′^ and ν_2_^′^ modes are 23 and 32 cm^–1^, respectively, and the off-diagonal anharmonicity (Δ_*ij*_) is ca. 38 cm^–1^. These values are fairly similar to those previously obtained for complex **H1** in DMF,^9^ and thus show that the mode (de)­localization and coupling strength are preserved upon a one-electron oxidation.

The second oxidation triggers a more significant change in the IR spectra (Figure 4). In this case, the ν_1_^′^ and ν_2_^′^ bands collapse into a strong, single band centered at ca. 2016 cm^–1^ (which we label as ν″). This change is in line with the expected deprotonation of the transiently generated **H1** ^2+^ species via PCET, yielding the 16 e^–^ square planar [Ir^I^(CO)­(PPh_3_)_3_]^+^ species (**1**_CO_^+^).

**Figure 4:**
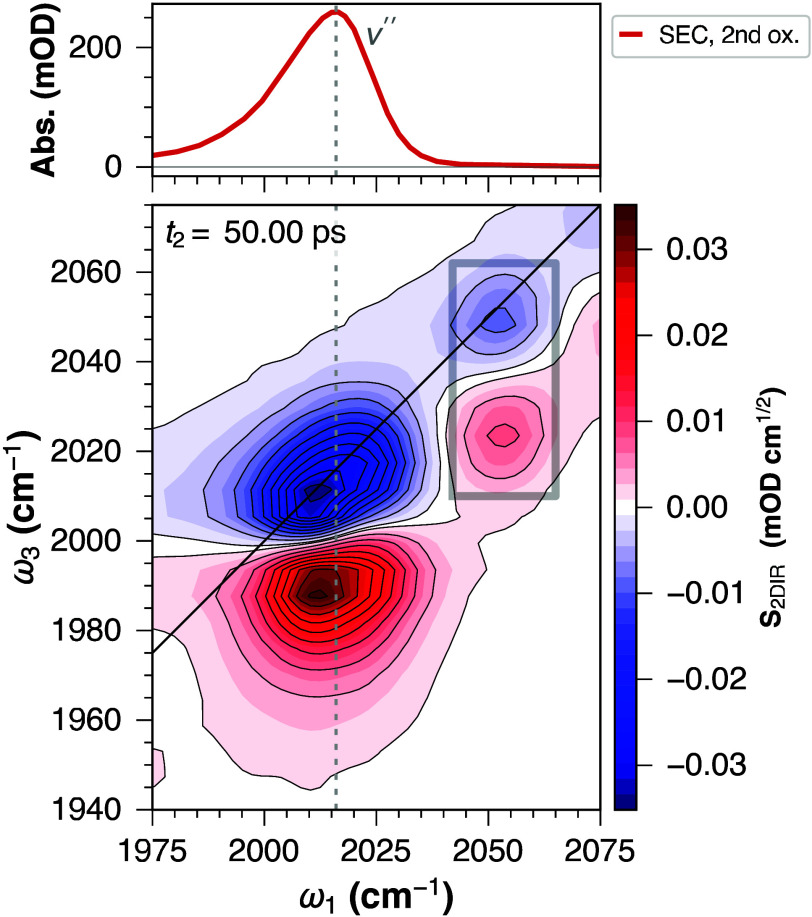
FT-IR SEC (*top*) and 2D-IR-SEC (*bottom*) spectra of **1**_CO_^+^ (at +280 mV vs Fc^+/0^ in DCM). The vertical dashed line connects the absorption and 2D-IR-SEC spectral features. The black square points to an unidentified band, present only in the 2D-IR-SEC spectra.

The significantly longer vibrational lifetime (in the order of ∼ 40 ps), lack of vibrational coupling, and apparent diagonal anharmonicity of 25 cm^–1^ suggest that this band corresponds to an isolated Ir­(C≡O) vibration, providing further evidence that this species is indeed the **1**_CO_^+^ complex. These spectral features are in agreement with our previous results obtained for the deuterated analogue of Vaska’s complex dihydrogen adduct, [(D)_2_Ir^III^(CO)­(PPh_3_)_2_Cl]; and for the HCl adduct of Vaska’s complex, *trans,cis*-[HIr^III^(CO)­(PPh_3_)_2_Cl_2_]—both complexes lacking vibrational coupling and delocalization of the Ir­(C≡O) stretching mode to a *trans* Ir–H ligand mode.^9^

The appearance of additional bands at ca. 2050 and 2075 cm^–1^ in the 2D-IR-SEC experiments at +280 mV vs Fc^+/0^ can be potentially attributed to impurities or side products from the electrochemical oxidation in the OTTLE cell due to the longer accumulation times required for the acquisition of 2D-IR-SEC spectra. These features are also observed (although to a much lesser extent) in the 2D-IR-SEC spectra collected at the potential of the first oxidation (Figure 3).

A closer inspection of the 2D-IR line shapes of the vibrational modes with a larger Ir–H character for both the parent (**H1**) and singly oxidized complexes (**H1** ^+^), i.e., ν_2_ and ν_2_^′^, respectively (Figures S3–S4 in the Supporting Information), reveals a significant homogeneous contribution with a slight inhomogeneity that decays within the vibrational lifetime (ca. 10 ps). A remaining, larger inhomogeneous contribution in the case of **H1** ^+^, extends beyond the vibrational lifetime. We believe that these subtle changes in spectral diffusion dynamics are tentatively attributable to the change in net charge of the complex (going from neutral to cationic), which in the electrolyte solution used for SEC experiments may involve a closer interaction between the complex and the electrolyte counterions.

Herein, we demonstrate that ultrafast 2D-IR-SEC provides unprecedented insight into the PCET reactivity of the hydridocarbonyl complex **H1**. The strong anharmonic coupling between the Ir–H and Ir­(C≡O) stretching vibrations allows simultaneous tracking of the metal oxidation state and hydride protonation state during electrochemical oxidation. While the first oxidation yields a stable 17 e^–^ species (**H1** ^+^), the second oxidation triggers irreversible deprotonation. This highlights the delicate balance between PCET and potential disproportionation pathways. Our work thus establishes 2D-IR-SEC as a powerful tool for elucidating PCET mechanisms in metal hydrides, with broad implications for catalytic fuel production. The approach demonstrated herein is readily applicable to other redox-active metal complexes, subject to the presence of vibrational couplings between the M–H and M­(C≡O) modes, offering a general strategy to disentangle coupled electron and proton transfer dynamics in real time.

## Supplementary Material





## Data Availability

The data supporting the findings of this study can be downloaded freely at DOI: 10.5281/zenodo.18435618.

## Notes

Full experimental details, including ultrafast 2D-IR methods, data analysis, and details of the computational calculations; and additional figures (PDF)
XYZ coordinates of all DFT-optimized structures (XYZ)
