# Supplementary material for: Proton-Coupled Electron Transfer from the Hydride Perspective: Resolving Metal Oxidation and Protonation in a Hydridocarbonyl Complex by 2D-IR Spectroelectrochemistry
Source: J Phys Chem Lett. 2026 Feb 25;17(10):3008–12. doi: 10.1021/acs.jpclett.5c03977 (PMC13047692; doi:10.1021/acs.jpclett.5c03977)
Supplement: Supplementary file 1 [file jz5c03977_si_001.pdf]

Supporting Information for:

Proton-Coupled Electron Transfer from the Hydride Perspective: Resolving Metal Oxidation and Protonation in a Hydridocarbonyl Complex by 2D-IR Spectroelectrochemistry

Ricardo J. Fernández-Terán,[ab]* Iona I. Ivalo,[b] Dimitri Chekulaev,[b] and Julia A. Weinstein[b]

[a] *Department of Physical Chemistry, University of Geneva. CH-1205, Geneva, Switzerland.*

[b] *Department of Chemistry, University of Sheffield. Sheffield S3 7HF, United Kingdom.*

**Corresponding Author:* Ricardo.FernandezTeran@unige.ch

(Dated: January 30, 2026)

Contents

Page

S1. Experimental S2

1.1. Chemicals and Solvents S2

1.2. Electrochemical Measurements S2

1.3. FT-IR and UV/Vis Spectroelectrochemistry S2

1.4. Ultrafast 2D-IR Spectroelectrochemistry S3

1.5. Computational Methods S4

S2. Additional Figures S4

S3. References S6

S1. Experimental

1.1 Chemicals and Solvents

The complex $[HIr(CO)(PPh_3)_3]$ (**H1**, Alfa-Aesar), and triphenylphosphine (PPh_3, Sigma-Aldrich) were used as received from their suppliers. $[Bu_4N][PF_6]$ (Fluorochem) was recrystallised from EtOH and oven-dried at 80 °C. Solvents for (spectro)electrochemical measurements were suitably dried and degassed (obtained at the Grubbs dry solvent facility of the University of Sheffield).

1.2 Electrochemical Measurements

Electrochemical measurements were performed in a low-volume one-compartment cell using a Metrohm AutoLab PGSTAT100 potentiostat. Experiments were carried out at room temperature under a N_2 atmosphere (presaturated with the solvent vapours). The complexes were dissolved to a low millimolar concentration (typically ca. 2 mM) using 0.4 M $[Bu_4N][PF_6]$ as supporting electrolyte in dry and degassed DCM. The electrochemical setup consisted of a glassy carbon disc (3 mm dia., CH Instruments), and a Pt wire, serving respectively as working and counter electrodes. Ag/AgCl (3 M aq. KCl) was used as reference electrode. Potentials were referenced to the $Fc^{+/0}$ redox couple, added as an internal reference.

1.3 FT-IR and UV/Vis Spectroelectrochemistry

Fourier-transform infrared spectroelectrochemistry (FT-IR-SEC) was performed using an optically-transparent thin layer electrochemical (OTTLE) cell,[1] connected to the potentiostat described in the previous section. IR spectra were collected in a PerkinElmer One Spectrometer equipped with a room-temperature DTGS detector, with a 2 cm^{-1} resolution. The cell consisted of two 5 mm thick CaF_2 windows separated by a ca. 200 μm polypropylene spacer, having Pt mesh working and counter electrodes, and a silver wire as pseudo-reference electrode. The complexes were dissolved to ca. 2.5 mM concentrations (with 3 eq. of PPh_3) in 0.4 M $[Bu_4N][PF_6]$ in dry and degassed DCM. The electrolyte solution was taken as the background, and potential-dependent spectra were collected after equilibration of the applied potential for 3.5 min (spectral acquisition time of $\sim$1.5 min, 5 min per potential step in total). The potentials were referenced to the $Fc^{+/0}$ couple, added as an internal reference after the spectral measurements.

UV/Vis spectroelectrochemistry (UV/Vis-SEC) measurements were performed using the same potentiostat and OTTLE cell described above, in a Varian/Agilent Cary 5000 UV/Vis spectrophotometer. The complexes were dissolved to approx. 2.5 mM concentrations (with 3 eq. of PPh_3) in 0.4 M $[Bu_4N][PF_6]$ in dry and degassed DCM. A spectrum of the electrolyte solution was taken as the background, and potential-dependent spectra were collected after equilibration of the applied potential for 3.5 min (spectral acquisition time of ca. 1.5 min, for a total of 5 min per potential step). The potentials were referenced as described in the previous section.

1.4 Ultrafast 2D-IR Spectroelectrochemistry

The setup used in this work is part of the Lord Porter Laser Facility at the University of Sheffield. In brief, a Ti:Sapphire amplifier system (Spectra Physics Spitfire ACE PA-40), producing 800 nm, $\sim$40 fs pulses at 10 kHz, was used to pump an optical parametric amplifier (TOPAS, Light Conversion), which provided tunable mid-IR pulses centred at 2050 cm^{-1} (FWHM of $\sim$300 cm^{-1}) by difference frequency generation in $AgGaS_2$. After the OPA, the beam was passed through a reflective telescope, and a 95:5 BaF_2 wedge beam splitter was used to generate the mid-IR pump and probe pulses, respectively. The pump pulse pairs were obtained from a commercially available pulse shaper (QuickShape IR, PhaseTech).[2–4] The probe and reference pulses were obtained by further splitting the probe branch using a 50:50 Ge beam splitter, and the reflected beam was taken as the probe. All pulses were focussed by an off-axis parabolic mirror ($f = 21.1$ cm), and the pump and probe beams were overlapped at the sample position. The probe and reference beams were collimated after the sample by an identical parabolic mirror, and both were split once more by a 50:50 Ge beam splitter to generate two independent copies, which were then focussed into two separate spectrographs (Horiba iHR320, with 50 or 100 l mm^{-1} gratings), and detected using two LN_2-cooled multichannel HgCdTe detectors, with 96 probe pixels and 32 reference pixels each (Infrared Associates).

Broadband IR pump–IR probe experiments were performed with two-frame phase cycling and chopping. Fully absorptive 2D-IR spectra were recorded in the time domain with a dual rotating frame ($\omega_r = 1200$ and -250 cm^{-1} for the fixed and scanned pump pulses, respectively; total 1450 cm^{-1}). The coherence (t_1) delays were scanned up to 4 ps with undersampling ($\Delta t_1 = 15$ fs). A four-frame phase cycling scheme[2,3] was adopted to remove pump/probe scatter ($I_{1,x}$ and $I_{2,x}$, with $x = 3$ or LO), and the residual pump/pump ($I_{1,2}$) scatter was removed by subtraction of a 2D-IR spectrum collected at a very negative delay ($t_2 = -50$ ps). In all experiments, dispersion compensation up to third order and active Bragg angle correction were used to compress the IR pump pulses.[5,6] A multichannel referencing scheme, as described by Ge and co-workers,[7,8] was used for noise suppression.

The data were apodised with a cosine function and zero-padded ($\times 2$), then Fourier transformed to reveal the frequency-resolved 2D-IR spectra, yielding an effective resolution of 2.2 cm^{-1} along ω_1, and average resolutions of 5.8 and 2.8 cm^{-1} along ω_3 (depending on the grating). Calibration of the detector and shaper frequency axes was achieved by fitting the central wavelength and resolution to an experimental FT-IR spectrum of neat 1,4-dioxane in a 100 μm cell. Typical IR pump energies were in the order of 1.5 μJ at the sample position, with a spot size of ca. 350 μm for the pump and 300 μm for the probe. All pulses were *p*-polarised.

The sample was prepared as described for FT-IR spectroelectrochemistry, but with a higher concentration of the complex (ca. 20 mM) and a large excess of PPh_3. A modified version of the OTTLE cell described above was used for the ultrafast experiments, with thinner CaF_2 windows (2 mm), a 150 μm thick polypropylene spacer, and a 3$\times$3 mm square hole in the centre of the working electrode (to avoid pump scatter). The cell was connected to a portable potentiostat (PalmSens EmStat3) and the corresponding potential was applied until equilibration (typically 10–15 min), monitored by following the live IR pump–IR probe signal.

1.5 Computational Methods

Density functional theory (DFT) calculations were performed using Gaussian 16 rev. A.03[9] using the B3LYP functional, the def2-SVP basis set (for all atoms) and def2-ECP effective core potential (for Ir).[10,11] The IEF-PCM solvation model (as implemented in Gaussian) was used for calculations in DCM solution,[12] together with Grimme's D3 dispersion correction.[13] The structures were optimised with tight convergence criteria and an ultra-fine integration grid. Harmonic vibrational analysis revealed no negative frequencies, confirming them to be true minima.

S2. Additional Figures

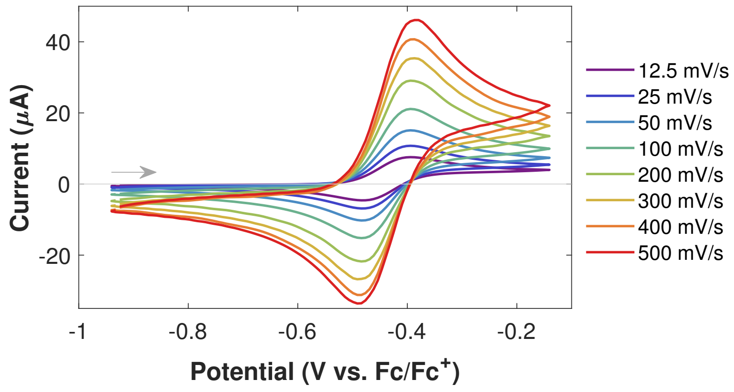

Figure S1: Cyclic voltammograms of **H1** (2 mM, with 5 eq. PPh_3) in 0.4 M $[Bu_4N][PF_6]$ in DCM as a function of scan rate. Potentials scanned up to the first oxidation, showing a reversible diffusion-controlled behaviour. An arrow indicates the scan direction.

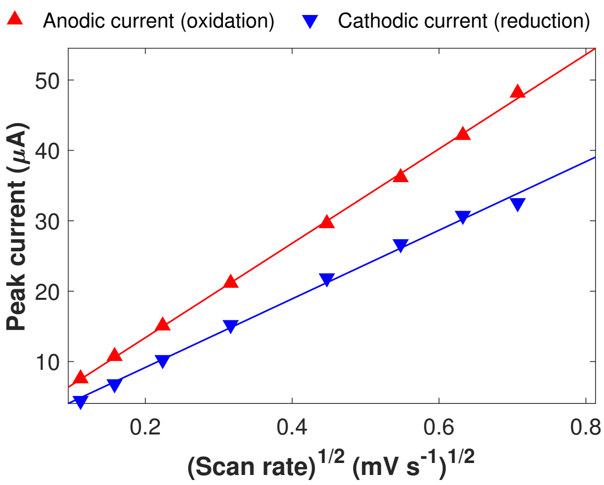

Figure S2: Randles–Ševčík plot of the absolute value of the peak anodic or cathodic current vs. square root of the scan rate for the CVs shown in Figure **S1**. The linear trends show that the first oxidation process in **H1** is diffusion-controlled and reversible.

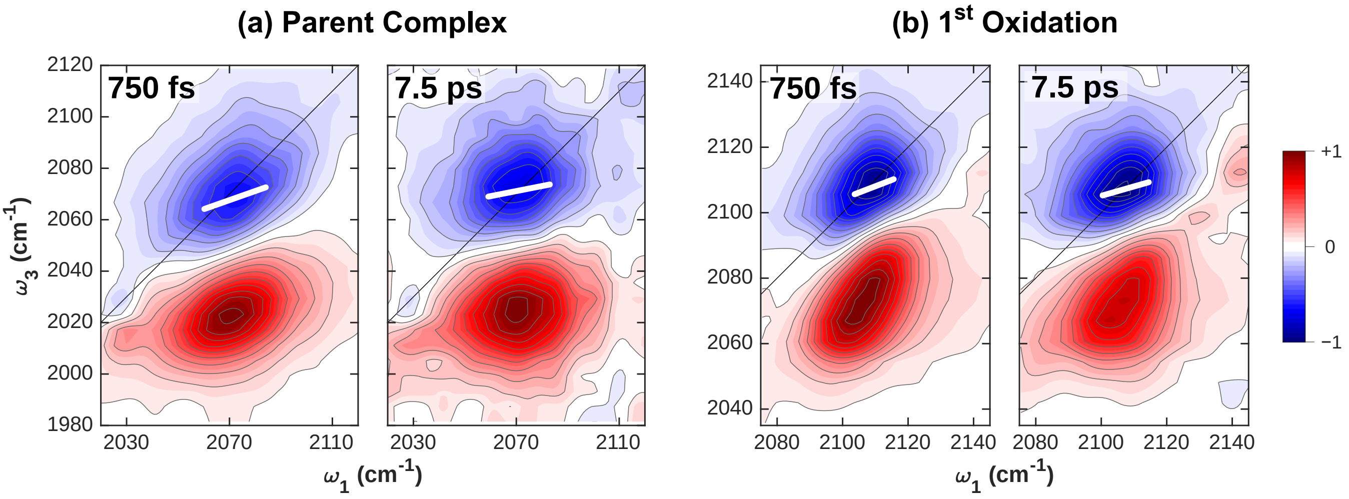

Figure S3: 2D-IR(-SEC) spectra (close-up of the ν_2 and ν_2' vibrational bands) of complexes **H1** (*left panel*) and **H1**$^+$ (at –380 mV vs Fc$^{+/0}$, *right panel*) at early and late population delays. White lines indicate the centerline slopes, used as a metric for spectral diffusion.

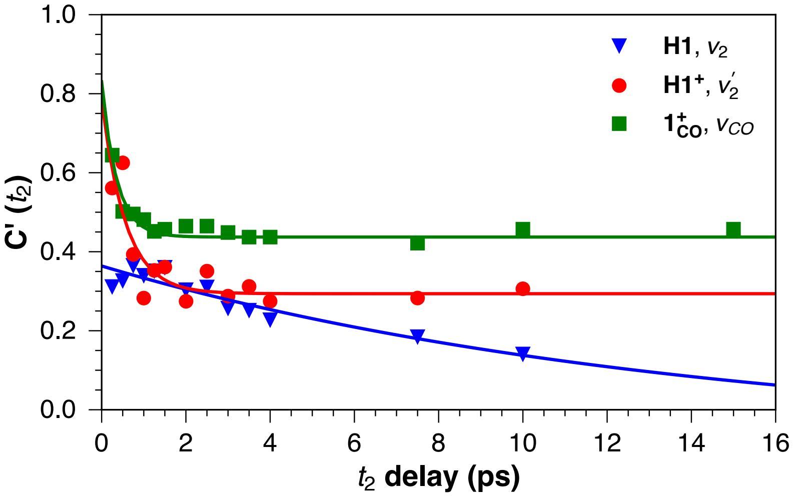

Figure S4: Centerline slope values $[C'(t_2)]$ obtained after analysis of the datasets corresponding to the ν_2 band of **H1** (blue), the ν_2' band of **H1**$^+$ (red), and the ν_{CO} band of $\mathbf{1}^+_{\mathrm{CO}}$. The solid lines represent fits to an exponential decay plus offset, given by $C'(t_2) = a_0 + a_1 \exp\left(-t_2/\tau_1\right)$.

S3. References

(1) M. Krejčik, M. Daněk, and F. Hartl, Simple construction of an infrared optically transparent thin-layer electrochemical cell. Applications to the redox reactions of ferrocene, $Mn_2(CO)_{10}$ and $Mn(CO)_3$(3,5-di-t-butyl-catecholate)$^-$, J. Electroanal. Chem. Interfacial Electrochem. **317**, 179 (1991).

(2) S. H. Shim and M. T. Zanni, How to turn your pump–probe instrument into a multidimensional spectrometer: 2D IR and Vis spectroscopies via pulse shaping, Phys. Chem. Chem. Phys. **11**, 748 (2009).

(3) C. T. Middleton, A. M. Woys, S. S. Mukherjee, and M. T. Zanni, Residue-specific structural kinetics of proteins through the union of isotope labeling, mid-IR pulse shaping, and coherent 2D IR spectroscopy, Methods **52**, 12 (2010).

(4) A. Ghosh, A. L. Serrano, T. A. Oudenhoven, J. S. Ostrander, E. C. Eklund, A. F. Blair, and M. T. Zanni, Experimental implementations of 2D IR spectroscopy through a horizontal pulse shaper design and a focal plane array detector, Opt. Lett. **41**, 524 (2016).

(5) J. M. Nite, J. D. Cyran, and A. T. Krummel, Active Bragg angle compensation for shaping ultrafast mid-infrared pulses, Opt. Express **20**, 23912 (2012).

(6) A. C. Jones, M. B. Kunz, I. Tigges-Green, and M. T. Zanni, Dual spectral phase and diffraction angle compensation of a broadband AOM 4-f pulse-shaper for ultrafast spectroscopy, Opt. Express **27**, 37236 (2019).

(7) Y. Feng, I. Vinogradov, and N.-H. Ge, General noise suppression scheme with reference detection in heterodyne nonlinear spectroscopy, Opt. Express **25**, 26262 (2017).

(8) Y. Feng, I. Vinogradov, and N.-H. Ge, Optimized noise reduction scheme for heterodyne spectroscopy using array detectors, Opt. Express **27**, 20323 (2019).

(9) M. J. Frisch, G. W. Trucks, H. B. Schlegel, G. E. Scuseria, M. A. Robb, J. R. Cheeseman, G. Scalmani, V. Barone, G. A. Petersson, H. Nakatsuji, X. Li, M. Caricato, A. V. Marenich, J. Bloino, B. G. Janesko, R. Gomperts, B. Mennucci, H. P. Hratchian, J. V. Ortiz, A. F. Izmaylov, J. L. Sonnenberg, D. Williams-Young, F. Ding, F. Lipparini, F. Egidi, J. Goings, B. Peng, A. Petrone, T. Henderson, D. Ranasinghe, V. G. Zakrzewski, J. Gao, N. Rega, G. Zheng, W. Liang, M. Hada, M. Ehara, K. Toyota, R. Fukuda, J. Hasegawa, M. Ishida, T. Nakajima, Y. Honda, O. Kitao, H. Nakai, T. Vreven, K. Throssell, J. Montgomery, J. A., J. E. Peralta, F. Ogliaro, M. J. Bearpark, J. J. Heyd, E. N. Brothers, K. N. Kudin, V. N. Staroverov, T. A. Keith, R. Kobayashi, J. Normand, K. Raghavachari, A. P. Rendell, J. C. Burant, S. S. Iyengar, J. Tomasi, M. Cossi, J. M. Millam, M. Klene, C. Adamo, R. Cammi, J. W. Ochterski, R. L. Martin, K. Morokuma, O. Farkas, J. B. Foresman, and D. J. Fox, Gaussian 16, Revision A.03 (2016).

(10) F. Weigend and R. Ahlrichs, Balanced basis sets of split valence, triple zeta valence and quadruple zeta valence quality for H to Rn: Design and assessment of accuracy, Phys. Chem. Chem. Phys. **7**, 3297 (2005).

(11) F. Weigend, Accurate Coulomb-fitting basis sets for H to Rn, Phys. Chem. Chem. Phys. **8**, 1057 (2006).

(12) G. Scalmani and M. J. Frisch, Continuous surface charge polarizable continuum models of solvation. I. General formalism, J. Chem. Phys. **132**, 114110 (2010).

(13) S. Grimme, J. Antony, S. Ehrlich, and H. Krieg, A consistent and accurate ab initio parametrization of density functional dispersion correction (DFT-D) for the 94 elements H-Pu, J. Chem. Phys. **132**, 10.1063/1.3382344 (2010).
